# Association between circulating alpha-1 antitrypsin polymers and lung and liver disease

**DOI:** 10.1186/s12931-021-01842-5

**Published:** 2021-09-15

**Authors:** Alexa Núñez, Irene Belmonte, Elena Miranda, Miriam Barrecheguren, Georgina Farago, Eduardo Loeb, Mònica Pons, Francisco Rodríguez-Frías, Pablo Gabriel-Medina, Esther Rodríguez, Joan Genescà, Marc Miravitlles, Cristina Esquinas

**Affiliations:** 1 Pneumology Department, Hospital Universitari Vall d´Hebron, Vall d’Hebron Institut de Recerca (VHIR), Vall d’Hebron Barcelona Hospital Campus, P. Vall d’Hebron 119-129, 08035 Barcelona, Spain; 2 Universitat Autònoma de Barcelona, Bellaterra, 08193 Barcelona, Spain; 3 Department of Biology and Biotechnologies, ‘Charles Darwin’ and Pasteur Institute - Cenci Bolognetti Foundation, Sapienza University of Rome, Rome, Italy; 4 Pneumology Department, Teknon Medical Center, Barcelona, Spain; 5 Liver Unit, Department of Internal Medicine, Hospital Universitari Vall d’Hebron, Vall d’Hebron Institut de Recerca (VHIR), Vall d’Hebron Barcelona Hospital Campus, Barcelona, Spain; 6 Department of Clinical Biochemistry, Hospital Universitari Vall d’Hebron, Vall d’Hebron Barcelona Hospital Campus, Barcelona, Spain; 7 Centro de Investigación Biomédica en Red de Enfermedades Hepáticas y Digestivas, (CIBEREHD), Barcelona, Spain; 8 Clinical Biochemistry Research Group/Vall d’Hebron Institut de Recerca (VHIR), Vall d’Hebron Barcelona Hospital Campus, Barcelona, Spain; 9 Centro de Investigación Biomédica en Red de Enfermedades Respiratorias (CIBERES), Barcelona, Spain

**Keywords:** Alpha-1 antitrypsin deficiency, Circulating polymers, Emphysema, Liver disease

## Abstract

**Background:**

Alpha-1 antitrypsin deficiency (AATD) is considered one of the most common genetic diseases and is characterised by the misfolding and polymerisation of the alpha-1 antitrypsin (AAT) protein within hepatocytes. The relevance of circulating polymers (CP) of AAT in the pathogenesis of lung and liver disease is not completely understood. Therefore, the main objective of our study was to determine whether there is an association between the levels of CP of AAT and the severity of lung and liver disease.

**Method:**

This was a cross-sectional study in patients with different phenotypes of AATD and controls. To quantify CP, a sandwich ELISA was performed using the 2C1 monoclonal antibody against AAT polymers. Sociodemographic data, clinical characteristics, and liver and lung parameters were collected.

**Results:**

A cohort of 70 patients was recruited: 32 Pi*ZZ (11 on augmentation therapy); 29 Z-heterozygous; 9 with other genotypes. CP were compared with a control group of 47 individuals (35 Pi*MM and 12 Pi*MS). ZZ patients had the highest concentrations of CP (p < 0.001) followed by Z heterozygous. The control group and patients with Pi*SS and Pi*SI had the lowest CP concentrations. Pi*ZZ also had higher levels of liver stiffness measurements (LSM) than the remaining AATD patients. Among patients with one or two Z alleles, two patients with lung and liver impairment showed the highest concentrations of CP (47.5 µg/mL), followed by those with only liver abnormality (n = 6, CP = 34 µg/mL), only lung (n = 18, CP = 26.5 µg/mL) and no abnormalities (n = 23, CP = 14.3 µg/mL). Differences were highly significant (p = 0.004).

**Conclusions:**

Non-augmented Pi*ZZ and Z-patients with impaired lung function and increased liver stiffness presented higher levels of CP than other clinical phenotypes. Therefore, CP may help to identify patients more at risk of developing lung and liver disease and may provide some insight into the mechanisms of disease.

## Background

Alpha-1 antitrypsin deficiency (AATD) is considered one of the most common genetic disorders in adults [1], with a prevalence of 1 in 2000 to 3000 live births in Europe [2]. AATD is characterised by low circulating levels of the alpha-1 antitrypsin (AAT) protein caused by specific mutations in the *SERPINA1* gene resulting in a misfolded protein and intracellular liver polymerization. *SERPINA1* is highly polymorphic with at least 120 mutations having been described, and of these, 60 are deficient variants [3, 4]. The normal allele present in 95% of healthy individuals is defined as the M allele, and the most common deficient variants are S and Z [5, 6]. The alleles of *SERPINA1* are codominant, therefore, individuals heterozygous and homozygous for the Z allele have AAT plasma concentrations of 50% and 10 to 15% of normal, respectively.

In normal conditions, the protein is mainly synthesised and secreted by hepatocytes and its main function is to protect lung tissue from damage caused by proteolytic enzymes such as neutrophil elastase. In the presence of the Z allele, most of the AAT synthesised polymerises and accumulates in the lumen of the endoplasmic reticulum (ER) of liver cells as inclusion bodies. These inclusions are associated with neonatal hepatitis, cirrhosis and hepatocellular carcinoma [7]. In addition, lower concentrations of circulating AAT predispose to early onset panlobular emphysema in individuals with smoking history [8–10].

Polymers of AAT have also been identified in the bronchoalveolar lavage fluid and alveolar walls of carriers of the Z allele [11]. In a study by Alam et al. [12], it was observed that cigarette smoking accelerated polymerisation of AAT in patients homozygous for the Z allele, leading to a greater depletion of the protection against neutrophil elastase in the lung. Later, other authors found that these circulating polymers (CP) of AAT were present in serum samples of AATD Pi*ZZ and mixed phenotypes patients, probably due to secretion to the circulation from liver cells [13, 14].

However, little is known about the role of CP of AAT in the pathogenesis of disease in patients with AATD. Therefore, the aim of our study was to determine CP concentrations in individuals with different genotypes of AAT and to investigate the association between CP and the severity of lung and liver disease in patients with AATD homozygous and heterozygous for the Z allele.

## Methods

This was a cross-sectional study performed in the Vall d’Hebron Hospital Campus (Barcelona, Spain), which is a reference centre for AATD [15]. Patients with moderate and severe deficiency (genotypes Pi*SS, MZ, SZ, ZZ and rare variants) were consecutively included from the AATD outpatient clinic of the Pneumology Department between January and December 2019. A control group of adults, older than 18 years with Pi*MM and Pi*MS genotypes were also consecutively recruited during the same period among those attending routine medical check-ups in the Preventive Medicine outpatient clinic in our centre.

The study was carried out according to the principles of the Declaration of Helsinki and the prevailing norms for performing investigation in humans. Data confidentiality was ensured according to the Law of Data Protection 2016/679. The study was approved by the Ethical Committee and Clinical Investigation of the Vall d’Hebron University Hospital (Barcelona, Spain) number PR (AG) 156/2016, and all the participants provided written informed consent.

### Variables

Sociodemographic data and clinical characteristics were collected from all patients. Comorbidities were registered according to the Charlson comorbidity index [16]. Patients performed spirometry and values for forced expiratory volume in the 1st second (FEV_1_), forced vital capacity (FVC) and the FEV_1_/FVC ratio were registered. Chronic obstructive pulmonary disease (COPD) was diagnosed when the post-bronchodilator FEV_1_/FVC ratio was below 0.7.

Liver stiffness measurement (LSM) was performed using transient elastography (Fibroscan 502 Touch, Echosens, Paris, France) in a fasting state according to the usual standard procedure [17]. Quality criteria were at least 10 valid measurements and an interquartile to median ratio ≤ 30%. Only valid assessments were considered for the analysis. Data were expressed in kilopascals (kPa). Normal LSM values vary between 4–6 kPa. LSM ≥ 6 kPa were considered abnormal and suggestive of liver disease/mild fibrosis.

### Laboratory testing

Biochemical tests included determination of liver enzymes: aspartate-aminotransferase (AST), alanine-aminotransferase (ALT), gamma-glutamyl transferase and alkaline phosphatase. In addition, two fibrosis biomarkers were assessed: the fibrosis-4 (FIB-4) score and the enhanced liver fibrosis (ELF) test. The FIB-4 score was calculated as age (years) × AST [IU/L]/(platelet count [10^9^/L] × √ALT [IU/L]). The ELF test (Siemens Healthcare Diagnostics, Vienna, Austria) consists of three components: type III procollagen peptide, hyaluronic acid and tissue inhibitor of metalloproteinase-1 and is a marker of liver fibrosis [18]. In addition, fibrinogen and C-reactive protein (CRP) were determined as markers of systemic inflammation.

### AAT blood levels and genotyping

Quantitative measurement of AAT levels was determined by immune nephelometry and genotyping was performed using real-time polymerase chain reaction or sequencing the entire encoding region of the *SERPINA1* gene as previously described [15].

### Circulating polymers of AAT

To quantify CP, a sandwich ELISA with plasma samples was performed using the 2C1 monoclonal antibody (mAb) against AAT polymers [19]. Plates were coated overnight at room temperature with 50 µL/well of purified 2C1 mAb at 2 µg/mL. The next day, the plates were washed and incubated with 300 µL/well of blocking solution for 1 h. Standards and samples were diluted in blocking buffer, added to the plate and incubated for 2 h at room temperature. Bound polymers were detected with anti-total AAT 3C11 mAb labelled with horseradish peroxidase and incubated for 75 min, and its activity was subsequently measured in a plate reader at 450 nm using a 3,3′,5,5′-tetramethylbenzidine (TMB) substrate solution. CP (µg/mL) concentrations were determined by interpolation of absorbance values on the standard curve [20]. Monoclonal antibody 2C1 recognizes the pathological polymers formed by AAT, however, in samples with elevated AAT concentrations and the absence of polymers, a minimal amount of monomeric AAT binds to mAb 2C1 with low affinity, showing a weak background signal. In order to reduce this noise, the proportion of polymers versus the total levels of AAT (%) was determined in all samples together with total polymer concentrations (µg/mL).

### Statistical analysis

Qualitative variables were described with absolute frequencies and percentages. The description of quantitative variables was performed using the mean, standard deviation (SD), median and quartiles. The Kolmogorov–Smirnov test was used to assess the normality of distributions.

The sociodemographic, clinical characteristics and CP levels (µg/mL and %) were compared according to the genotypes. In the case of quantitative variables, ANOVA tests were carried out with Bonferroni correction for multiple comparisons. The Chi-squared test (Fisher test for frequencies < 5) was used for the comparison of categorical variables. Linear relationships between clinical variables and levels of CP were also analysed using the Pearson correlation coefficient.

For all the tests, p-values < 0.05 were considered statistically significant. The statistical package R Studio (V2.5.1) was used for the analyses.

## Results

### Characteristics of participants

A total of 70 patients with different AAT genotypes were included. Among them, 32 (46%) were homozygous Pi*ZZ, of whom 11 were on augmentation therapy; 29 (41%) were heterozygous for the Z allele (13 Pi*MZ, 13 Pi*SZ, 1 Pi*MmaltonZ, 1 Pi*PLowelZ, 1 Pi*FZ); 4 (6%) carriers of the S allele (3 Pi*SS, 1 Pi*SI); and 5 (7%) rare variants (1 Pi*MMmattawa, 2 Pi*MMmalton, 1 Pi*SMmalton and 1 Pi*MMvall d’Hebron) (Table 1). The control group consisted of 47 individuals with a mean age of 46 years (SD = 14.1) and 17 (36.2%) were male; 35 had a normal genotype Pi*MM and 12 had a Pi*MS genotype.

**Table 1:** Description of AAT variants identified in the study population

Variant	Codon change	Classification	Deficiency
M	–	Normal	Not deficient
Z	Glu342Lys	Deficient	Severe
S	Glu264Val	Deficient	Moderate
F	Arg223Cys	Deficient	Not deficient Reduced inhibitory activity
I	Arg39Cys	Deficient	Moderate
Mmalton	Phe52del (M_2_)	Deficient	Severe
Mvall d’hebron	Pro369Ser	Deficient	Moderate
Plowell	Asp256Val (M_3_)	Deficient	Severe
Q_0_mattawa	Leu353Phefs*24 (M_1val_)	Null	Severe

*AAT* alpha-1 antitrypsin, *AATD* alpha-1 antitrypsin deficiency

### Sociodemographic and clinical characteristics of patients according to the AATD genotype

Patients were divided into three groups according to their AATD genotype: (1) homozygous Pi*ZZ, (2) heterozygous for the Z allele, (3) others: Pi*SS, Pi*SI, Pi*MMmattawa, Pi*MMmalton, Pi*SMmalton and Pi*MMvall d’Hebron.

No differences were observed between groups in terms of age, body mass index or sex distribution (Table 2).

**Table 2:** Characteristics of the 70 patients included in the study

Variables	ZZ (n = 32)	Z- (n = 29)	Other (n = 9)	p value
Age, years	54.6 (15.4)	50.1 (13.5)	47 (17.6)	0.235
Sex, male (%)	20 (62.5)	16 (55.2)	3 (33.3)	0.297
BMI (kg/m^2^)	24.1 (3.2)	24.7 (3.5)	24.6 (4.2)	0.683
Smokers (%)	0	6 (20.7)	1 (11.1)	0.120
Ex-smokers (%)	18 (58.1)	14 (48.3)	4 (44.4)	
Never smokers (%)	13 (41.9)	9 (31)	4 (44.4)	
Tobacco exposure (pack-years)	22.7 (14.6)	32.4 (34.1)	34 (16.7)	0.360
COPD (%)	21 (65.6)	9 (31)	2 (22)	0.008
Charlson index	2.2 (1.6)	1.5 (1.3)	1.5 (1.9)	0.122
FVC (%)	83 (27)	100 (19)	104 (15)	0.014
FEV_1_ (%)	69 (32)	92 (31.3)	99 (15)	0.007
FEV_1_/FVC	63 (16)	71 (17)	76 (4.8)	0.028
LSM (kPa)	5.3 (1.2)	4.5 (1.2)	4.4 (0.9)	0.008
FIB-4	1.5 (0.9)	1.1 (0.4)	1.1 (0.6)	0.115
Augmentation therapy (%)	11 (34)	0	0	< 0.001
Haemoglobin (g/dL)	15.4 (1.4)	14.4 (1.2)	14.2 (1.2)	0.008
Leukocytes (× 10^9^/L)	7.6 (2.7)	7.6.(2.8)	7.1 (1.5)	0.922
Platelets (× 10^9^/L)	242 (71)	245 (54)	282 (51)	0.163
AST (IU/L)	29.9 (12.2)	24.4 (7.2)	29.6 (18.9)	0.129
ALT (IU/L)	30.4 (18.1)	25.8 (14.1)	24.9 (13.7)	0.392
ALP (IU/L)	81.6 (25.2)	78.6 (29.2)	80.5 (21.5)	0.819
GGT (U/L)	32.9 (16.1)	37.5 (51.5)	41.8 (18.8)	0.234
Proteins (g/dL)	7.1 (0.5)	7.3 (0.3)	7.2 (0.5)	0.489
AAT (mg/dL)	40 (36)	74 (25)	82 (22)	< 0.001
CRP (mg/L)	0.2 (0.2)	0.3 (0.6)	0.2 (0.2)	0.261
Fibrinogen (g/L)	3.7 (0.5)	3.8 (0.8)	3.9 (0.7)	0.353
ELF	8.7 (0.9)	8.1 (0.7)	8.6 (0.8)	0.004
AAT polymers (µg/mL)	37.4 (16.4)	13.5 (8)	2.3 (2.9)	< 0.001
AAT polymers, %	12.8 (7.2)	2.2 (2)	0.3 (0.5)	< 0.001

Values are mean (standard deviation) unless otherwise specified. *Patient group* ZZ: homozygous patients to the Z allele; -Z: heterozygous patients to the Z allele (Pi*MZ, Pi*SZ, Pi*MmaltonZ, Pi*PlowelZ, Pi*FZ); other: Pi*SS, Pi*SI, Pi*MMmattawa, Pi*MMmalton, Pi*SMmalton and Pi*MMvall d’hebron)

*BMI* body mass index, *COPD* Chronic obstructive pulmonary disease, *FVC *forced vital capacity, *FEV*_*1*_ forced expiratory volume in the first second, *FIB-4* fibrosis-4 score, *LSM* liver stiffness measurement, *FIB-4* fibrosis-4 score, *AST* aspartate aminotransferase, *ALT* alanine aminotransferase, *ALP* alkaline phosphatase, *GGT* gamma-glutamyl transferase, *AAT* alpha-1 antitrypsin, *CRP* C-reactive protein, *ELF* enhanced liver fibrosis test

Homozygous Pi*ZZ patients had a lower FVC% (p = 0.014), a lower FEV_1_% (p = 0.07) and higher percentage of COPD (p = 0.008) than the other genotypes. Moreover, Pi*ZZ individuals showed higher LSM (p = 0.018) and ELF levels (p = 0.004) compared to the remaining patients.

Regarding laboratory findings, as expected, Pi*ZZ patients presented lower AAT levels (p < 0.001). No significant differences were observed for leukocytes, platelets, liver enzymes, CRP, FIB-4 or fibrinogen concentrations among groups (Table 2).

### Circulating polymer concentrations in the different AATD genotypes

As a group, the Pi*ZZ patients presented higher concentrations of CP than heterozygous patients and those with other genotypes (Table 2).

**Fig. 1:**
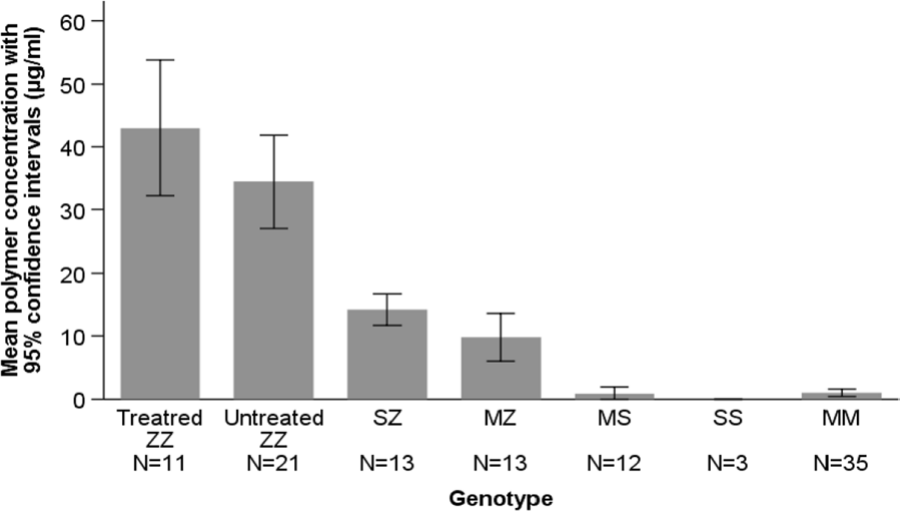
Concentrations of circulating polymers according to AATD genotypes. Differences between: treated and untreated Pi*ZZ p = 0.092; all Pi*ZZ vs. Pi*SZ p = 0.021; all Pi*ZZ vs. Pi*MZ p = 0.001; all Pi*ZZ vs. Pi*MS p < 0.001; all Pi*ZZ vs. Pi*SS p < 0.001; all Pi*ZZ vs. Pi*MM p < 0.001; Pi*SZ vs. Pi*MM p < 0.001; Pi*MZ vs. Pi*MM p = 0.001. (ANOVA with Bonferroni correction for multiple comparisons)

Considering the different genotypes individually, the highest values were observed in augmented Pi*ZZ patients (42.9 μg/mL (SD = 16) and one Pi*FZ with 42.1 μg/mL, very close to the 34.5 μg/mL (SD = 16.2) obtained in untreated Pi*ZZ patients. The lowest values were observed in controls (1.04 μg/mL (SD = 1.73) for Pi*MM and 0.9 μg/mL (SD = 1.7) for Pi*MS) and in patients with the Pi*SS and Pi*SI genotypes. Patients heterozygous for the Z allele and other rare variants had intermediate values (Table 3 and Fig. 1). The distribution of CP in percentage followed a similar distribution among genotypes (Table 3).

**Table 3:** Circulating polymer concentrations of the different AATD genotypes

AATD genotype	AAT polymers (µg/mL)	AAT polymers (%)	AAT (mg/dL)
Patients (n = 70)			
Pi*ZZ treated (n = 11)	42.9 (16)	9.2 (9.6)	73.5 (46.9)
Pi*FZ (n = 1)	42.1 (–)	6.2 (–)	67.7 (–)
Pi*ZZ untreated (n = 21)	34.5 (16.2)	14.7 (4.8)	23.1 (5.9)
Pi*MmaltonZ (n = 1)	22.8 (–)	10.2 (–)	22.2 (–)
Pi*PlowellZ (n = 1)	15.8 (–)	4.5 (–)	35.3 (–)
Pi*SZ (n = 13)	14.2 (4.2)	2.39 (0.6)	58.8 (8.3)
Pi*MZ (n = 13)	9.78 (6.3)	0.98 (0.5)	97 (16.5)
Pi*SMmalton (n = 1)	6.9 (–)	1.45 (–)	47.9 (–)
Pi*MMmattawa (n = 1)	5.5 (–)	0.8 (–)	66.9 (–)
Pi*MMvall d’hebron (n = 1)	4.1 (–)	0.3 (–)	126 (–)
Pi*MMmalton (n = 2)	2.3 (3.2)	0.2 (0.3)	80.7 (18.5)
Pi*SS (n = 3)	0	0	84.1 (10.5)
Pi*SI (n = 1)	0	0	85.0 (–)
Controls (n = 47)			
Pi*MM (n = 35)	1.04 (1.73)	0.06 (0.1)	172.7 (34.3)
Pi*MS (n = 12)	0.9 (1.7)	0.06 (0.1)	142.7 (20.1)

Values are mean (standard deviation)

*AATD* alpha-1 antitrypsin deficiency, *AAT* alpha-1 antitrypsin

### Correlation between circulating polymers and parameters of lung and liver impairment in untreated Pi*ZZ and Z- heterozygous patients

**Fig. 2:**
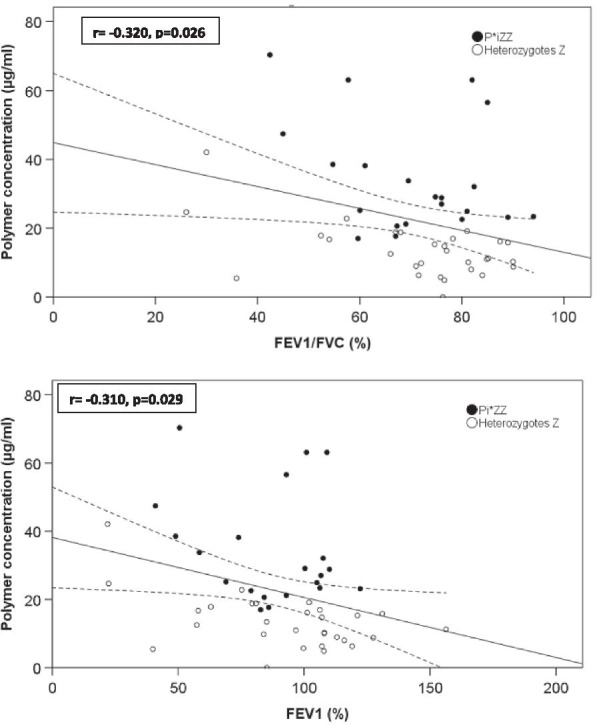
**a** Correlation between circulating polymers and FEV_1_/FVC in homozygous and heterozygous Z patients. **b** Correlation between circulating polymers and FEV_1_ (%) in homozygous and heterozygous Z patients. FEV_1_: forced expiratory volume in the 1st second; FVC: forced vital capacity; r indicates Pearson correlation coefficient; Linear regression fit (solid line) and 95% confidence interval (dashed line) of circulating polymer concentrations compared with FEV_1_/FVC and FEV_1_ (%) values

**Fig. 3:**
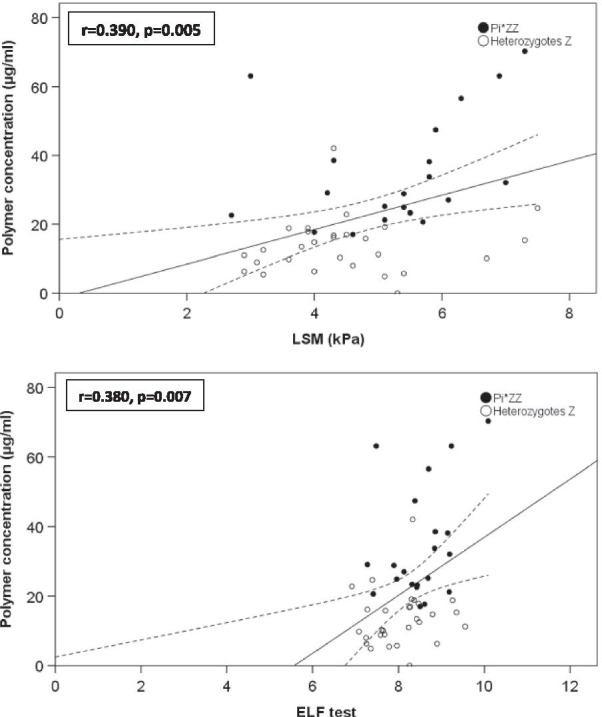
**a** Correlation between circulating polymers and LSM in homozygous and heterozygous Z patients. **b** Correlation between circulating polymers and ELF test in homozygous and heterozygous Z patients. LSM: liver stiffness measurements; r indicates Pearson correlation coefficient; Linear regression fit (solid line) and 95% confidence interval (dashed line) of polymer concentrations compared with LSM and ELF tests

In order to determine the possible relationship between CP concentrations and lung and liver alterations, we selected homozygous or heterozygous patients carrying the Z allele, excluding those on augmentation therapy. A negative, significant and weak linear relationship was found between CP concentrations and parameters of airflow obstruction; FEV_1_/FVC r = − 0.32, p = 0.026 and FEV_1_ (%) r = − 0.31, p = 0.029 (Fig. 2). Similarly, a positive and weak linear relationship was found between CP and LSM and ELF (r = 0.39 p = 0.005 and r = 0.38 p = 0.007, respectively) (Fig. 3). In contrast, correlations between AAT serum levels and lung and liver parameters were not significant (data not shown).

### Circulating polymer concentrations in patients according to the combined presence of lung and liver disease

The same group of unaugmented patients with one or two Z alleles was divided into four subgroups according to lung and liver impairment, using the cut-off of FEV_1_/FVC < 0.7 as diagnostic of COPD and LSM ≥ 6 kPa cut-off as suggestive of mild liver fibrosis. One patient was excluded from analysis due to missing data of LSM.

There was a gradient of CP concentrations, with the highest concentration in two patients with both lung and liver impairment (mean = 47.5 µg/mL), followed by six patients with liver abnormality only (mean CP = 34 µg/mL) and 18 with lung impairment only (mean CP = 26.5 µg/mL). Those with no abnormalities had the lowest CP concentrations (Table 4). Differences in CP concentrations between the four groups were significant (p = 0.004).

**Table 4:** Polymer concentrations and clinical characteristics: liver and/or lung disease

Patients heterozygous for the Z allele and untreated patients with the Pi*ZZ genotype (n = 49)				
	No COPD and LSM < 6 kPa (n = 23)	COPD and LSM < 6 kPa (n = 18)	No COPD and LSM ≥ 6 kPa (n = 6)	COPD and LSM ≥ 6 kPa (n = 2)
AAT (mg/dL)	61.1 (33.2)	39.4 (22.8)	47.5 (38.3)	76.1 (56.4)
AAT polymers (µg/mL)	14.4 (8)	26.5 (14.4)	34.0 (21.6)	47.5 (32.8)
AAT polymers (%)	4.7 (5.4)	9.8 (7.5)	11.2 (7.9)	10.8 (12.2)

Untreated patients with Pi*ZZ genotype (n = 21)				
	No COPD and LSM < 6 kPa (n = 6)	COPD and LSM < 6 kPa (n = 10)	No COPD and LSM ≥ 6 kPa (n = 4)	COPD and LSM ≥ 6 kPa (n = 1)
AAT (mg/dL)	19.4 (3.8)	22.4 (3.6)	27.4 (7.6)	36 (–)
AAT polymers (µg/mL)	25.3 (2.9)	32.3 (14.9)	44.7 (17.8)	70.3 (–)
AAT polymers (%)	13.2 (1.6)	14.5 (6.5)	16 (3.3)	19.4 (–)

COPD is defined as FEV_1_/FVC < 0.7

*LSM* liver stiffness measurement, *AAT* alpha-1 antitrypsin

The possible confounding effect of smoking was analysed, but there were only six current smokers among the 49 subjects (12.2%), being evenly distributed among the groups. Therefore, a possible confounding effect of smoking in our results can be reasonably ruled out.

## Discussion

Our results show that overall Pi*ZZ patients presented the highest levels of CP of AAT, followed by heterozygous Z patients and individuals with rare variants. The lowest CP concentrations were observed in controls with Pi*MM and Pi*MS genotypes and patients carrying the S allele, with undetectable levels in the few Pi*SS patients analysed. CP concentrations were significantly higher in patients with both lung and liver disease and correlated with the degree of alteration in lung function and liver stiffness.

Alpha-1 antitrypsin polymers are aggregates of misfolded protein and are deposited within the ER of hepatocytes, which is the basis of the pathogenesis of liver disease in AATD [7, 21]. Although most of the polymers remain as inclusion bodies in the ER of hepatocytes, some are secreted into the blood stream [13, 14]. Polymers are also secreted by alveolar macrophages and have a pro-inflammatory and chemotactic role for inflammatory cells in the lung. The polymers within alveolar macrophages have no anti-elastase activity, thereby contributing to a greater imbalance of the protease-antiprotease axis [22, 23]. Moreover, studies have shown that, apart from inactivating AAT by oxidation, cigarette smoke also increases the concentration of AAT polymers within alveolar macrophages [12, 24].

In our study, Z homozygous patients presented the highest concentrations of CP followed by Z heterozygous patients. These data were previously observed by Tan et al. [13], who reported the highest concentrations of CP in Pi*ZZ patients, a low signal in normal Pi*MM individuals and remained undetected in S-homozygous individuals. Other studies have also reported that, among the most frequent variants, the Z mutation polymerises the most and the S the least [21, 25, 26].

Patients with the Pi*ZZ genotype on augmentation therapy presented higher CP levels than untreated patients, despite blood samples being taken just before the following dose of augmentation therapy when plasma levels of exogenous AAT are minimal. This observation confirms previous studies that demonstrate the presence of AAT polymers in the augmentation therapy preparations [27] having a direct correlation with serum levels of AAT [28]. To avoid possible confounding effects caused by augmentation therapy, augmented patients were excluded from further analysis.

In order to assess the relationship between CP and variables of liver and lung disease we used data from untreated homozygous or heterozygous carriers of the Z allele. We found a negative relationship between CP concentrations and airflow obstruction parameters and a significant and positive linear relationship with LSM and ELF, suggesting that higher concentrations of CP are related to lung and liver damage. These findings are in agreement with a previous study on 244 Pi*ZZ individuals, that found a negative linear relationship between CP concentrations and the FEV_1_/FVC ratio. Moreover, although that study was not designed to assess liver disease, patients who self-reported abnormal liver function, liver disease or cirrhosis had higher CP concentrations than those without a history of liver involvement [13]. In a biopsy study, Mela et al. [29] found that higher polymer loads within hepatocytes were related to senescence of the cells and liver fibrosis. However, to the best of our knowledge, no other studies have related CP concentrations with LSM or ELF and this is important since transient elastography is increasingly used for the screening and follow-up of liver disease in patients with AATD [30, 31], and ELF is a systemic biomarker of liver fibrosis [18]. The importance of the polymerisation of mutated AAT in the pathogenesis of liver and lung disease in AATD has stimulated the development of new strategies of treatment for AATD based on the blockade of polymer formation [32, 33].

Our study has some limitations: (1) the cross-sectional design does not allow to establish a causal relationship between CP and liver and lung disease, and thus, longitudinal studies are necessary; (2) to demonstrate the impact of the load of CP on clinically relevant liver disease, a cut-off of LSM ≥ 6 kPa was used as suggestive of mild liver fibrosis. Although there is no validated cut-off of LSM for liver disease in AATD, we chose an arbitrary cut-off of LSM ≥ 6 kPa to be as sensitive as possible to detect cases of liver impairment [34–36]. (3) Finally, since AATD is a rare disease, we could not include enough patients with genotypes other than Z. Therefore, larger studies are required to demonstrate the importance of CP in the pathogenesis of lung and liver disease in rare variants. Data from large registries may help to identify patients with rare AATD variants [37, 38].

## Conclusions

Our results show that Pi*ZZ and heterozygous Z individuals present higher levels of CP than other AAT genotypes, and CP were associated with the presence and severity of lung and liver disease. Therefore, CP concentrations may help to identify AATD patients at greater risk of developing lung and liver disease and may provide some insight into the mechanisms of the disease. Larger studies are needed to demonstrate the importance of CP in other rare genotypes.

## Data Availability

Data are available from the authors under request.

## Abbreviations

AAT: Alpha-1 antitrypsin
AATD: Alpha-1 antitrypsin deficiency
ALAT: Alanine-aminotransferase
ANOVA: One-way analysis of variance
ASAT: Aspartate-aminotransferase
COPD: Chronic obstructive pulmonary disease
CP: Circulating polymers
CRP: C-reactive protein
ELF: Enhanced liver fibrosis
ELISA: Enzyme-Linked immunosorbent assay
ER: Endoplasmic reticulum
FEV1: Forced expiratory volume in one second
FIB-4: Fibrosis-4
FVC: Forced vital capacity
kPa: Kilopascals
LSM: Liver stiffness measurement
mAb: Monoclonal antibody
PI*: Protease inhibitor
SD: Standard deviation
TMB: 3,3′,5,5′-Tetramethylbenzidine
