# Correction to: Association between circulating alpha-1 antitrypsin polymers and lung and liver disease

**DOI:** 10.1186/s12931-021-01874-x

**Published:** 2021-11-01

**Authors:** Alexa Núñez, Irene Belmonte, Elena Miranda, Miriam Barrecheguren, Georgina Farago, Eduardo Loeb, Mònica Pons, Francisco Rodríguez-Frías, Pablo Gabriel-Medina, Esther Rodríguez, Joan Genescà, Marc Miravitlles, Cristina Esquinas

**Affiliations:** 1 Pneumology Department, Hospital Universitari Vall d´Hebron, Vall d’Hebron Institut de Recerca (VHIR), Vall d’Hebron Barcelona Hospital Campus, P. Vall d’Hebron 119-129, 08035 Barcelona, Spain; 2 Department of Medicine, Universitat Autònoma de Barcelona, Bellaterra, 08193 Barcelona, Spain; 3 Department of Biology and Biotechnologies, ‘Charles Darwin’ and Pasteur Institute - Cenci Bolognetti Foundation, Sapienza University of Rome, Rome, Italy; 4 Pneumology Department, Teknon Medical Center, Barcelona, Spain; 5 Liver Unit, Department of Internal Medicine, Hospital Universitari Vall d’Hebron, Vall d’Hebron Institut de Recerca (VHIR), Vall d’Hebron Barcelona Hospital Campus, Barcelona, Spain; 6 Department of Clinical Biochemistry, Hospital Universitari Vall d’Hebron, Vall d’Hebron Barcelona Hospital Campus, Barcelona, Spain; 7 Centro de Investigación Biomédica en Red de Enfermedades Hepáticas y Digestivas, (CIBEREHD), Barcelona, Spain; 8 Clinical Biochemistry Research Group/Vall d’Hebron Institut de Recerca (VHIR), Vall d’Hebron Barcelona Hospital Campus, Barcelona, Spain; 9 Centro de Investigación Biomédica en Red de Enfermedades Respiratorias (CIBERES), Barcelona, Spain

## Correction to: Respir Res (2021) 22:244 https://doi.org/10.1186/s12931-021-01842-5

The original version of this article [1] unfortunately contained a mistake.

The department details for second affiliation were missing in the original publication of the article. It has been added in this correction.

The correct affiliation details should read as

Department of Medicine, Universitat Autònoma de Barceona, Bellaterra, 08193 Barcelona, Spain.
